# Audit of Operative Notes Against Royal College of Surgeons Guidelines in a Tertiary Health Care Surgical Unit in Lahore

**DOI:** 10.7759/cureus.29313

**Published:** 2022-09-19

**Authors:** Aizaz Khalid, M. Zain ul Abideen Shahzad, Hamza Ahmed, Aima Gilani, Khalid H Khan

**Affiliations:** 1 Medicine and Surgery, Services Hospital Lahore, Lahore, PAK; 2 Surgery, Hameed Latif Hospital, Lahore, Lahore, PAK; 3 Orthopedics, Arif Memorial Teaching Hospital, Lahore, PAK; 4 Medicine and Surgery, Combined Military Hospital (CMH) Lahore, Lahore, PAK; 5 Diagnostic Radiology, The University of Lahore, Lahore, PAK; 6 Orthopedics and Spine Surgery, Rashid Latif Medical College, Arif Memorial Teaching Hospital, Lahore, PAK

**Keywords:** surgical practice, quality improvement projects, rcs guidlines, operative notes, clincal audit

## Abstract

Operative notes are important documentation made by the surgical team after a surgical procedure. They outline what happened during the surgery and several aspects of post-operative instructions. The aim of this audit was to compare current practices regarding documentation of these operative notes in a tertiary care hospital in Lahore. The standards used were the recommendations made in the good surgical practice guidelines by the Royal College of Surgeons (RCS) of England in 2014.

A prospective closed-loop audit was conducted using a checklist constructed using the RCS guidelines. In the first audit, which was done to analyze current practices, 44 consecutive handwritten operative notes were evaluated. The findings of this initial audit were presented to the surgical team in a local meeting and recommendations were made. Forty-eight consecutive operative notes were then analyzed in the re-audit done after an interval of one month. The findings of the re-audit were presented to the surgical team.

An overall 35.7% improvement was noted in the documentation of operative notes after the completion of the audit cycle. Certain parameters analyzed, such as thromboprophylaxis, antibiotic prophylaxis, and anticipated blood loss, were much more frequently documented in the re-audit.

In our study, implementation of recommendations such as restructuring of the operative notes pro forma and improving awareness regarding the RCS guidelines significantly improved the quality of operative notes that were being documented. Such audits should be conducted regularly to maintain and improve the standards of documentation in all surgical units.

## Introduction

Effective medical record keeping is a pivotal component of patient management. It helps in the scientific evaluation of the patient profiles, aiding in the analysis and formulation of treatment plans. It is also essential in current practice with regard to the issue of medical negligence [1]. Operative notes are one of the most important medical records in a surgical unit. Maintaining a full and proper record of operative notes is the professional responsibility of every surgeon [2]. A comprehensive record serves to streamline the patient management approach of the entire surgical team. It also serves as an effective defense in medico-legal cases where illegible or incomplete notes can weaken the surgeons' case.

Operative notes are one of the first medical records that are analyzed post-operatively by the critical care and surgical teams. They serve to orient healthcare professionals regarding the details of the procedure as well as essential post-operative care that the patient needs. Surgeons are directly involved in ascertaining the need of certain interventions such as thromboprophylaxis and antibiotics. These crucial decisions are made by surgeons on a case-to-case basis, and the post-operative team often seeks guidance regarding such management in the post-operative notes. Hence, the quality of operative notes is directly related to the effective management of the surgical patient.

In 2014, the Royal College of Surgeons of England introduced good surgical practice guidelines, which outline information that should be present in operative notes [3]. These guidelines are considered the standard against which the performance of several institutes has been audited previously [4-9]. The purpose of this audit was to evaluate the quality of operative notes against this recognized standard, identify pitfalls and make recommendations to improve operative notes in the Department of Surgery at Arif Memorial Hospital, Lahore.

## Technical report

Methods

This study was conducted in the Department of Surgery in Arif Memorial Hospital, Lahore. The approval for this audit was sought and granted in March 2022 by the local committee. All data were collected prospectively using a checklist that evaluated 20 parameters. These parameters were based on the 2014 Royal College of Surgeons of England's good surgical practice guidelines [3]. Operative notes were documented on a pro forma in Arif Memorial Hospital, Lahore. The data on the current practices were collected in March 2022. The data were analyzed and presented to the local committee in April 2022. Possible changes were also discussed and implemented following the meeting. The second set of data were collected in May-June 2022, the results of which were presented to the local meeting in July 2022 after analysis. All data were analyzed using SPSS Version 28.0.0.1 (IBM SPSS, Armonk, New York).

Data extracted from the pro forma was in the form of present, absent, or not applicable. Conditions of non-application of parameters were discussed in the local meeting preceding the first round of data collection. The checklist assessed the patient's information, date of procedure, time of the procedure, elective/emergency surgery, names of the operating surgeon and assistant, name of the theater anesthetist, the operative procedure carried out, incision, operative diagnosis, operative findings, any problems/complications, any extra procedure performed and the reason why it was performed, details of tissue removed/added or altered, details of closure technique, anticipated blood loss, antibiotic prophylaxis (where applicable), deep vein thrombosis (DVT) prophylaxis (where applicable), detailed post-operative care instructions, and signature and legibility of written operative notes.

Results

Forty-four operative notes were analyzed prospectively against the checklist in the initial audit conducted in March 2022 (Table 1). Patient identification, date of procedure, and name of the surgeon were documented in 97.7% (43), making them the most documented parameters. DVT prophylaxis was not documented in any of the 44 notes. Anticipated blood loss and antibiotic prophylaxis were documented in 2.3% (1) operative notes, making them the least documented parameters. About 93.2% (41) of the notes were deemed to be satisfactorily legible and 6.8% (3) were poorly legible. Overall, 58.6% of all parameters were documented in these operative notes.

**Table 1:** Frequency of documentation of the parameters identified in the Royal College of Surgeons guidelines. DVT: deep vein thrombosis.

Parameter assessed	Frequency of documentation in the initial audit cycle (n)	Frequency of documentation in the re-audit cycle (n)	Change
Patient identification	97.7% (43)	91.7% (44)	−6%
Date of procedure	97.7% (43)	100% (48)	2.3%
Time of procedure	95.5% (42)	87.5% (42)	−8%
Elective/emergency procedure	15.9% (7)	100% (48)	84.1%
Name of surgeon and assistant	97.7% (43)	100% (48)	2.3%
Names of theater anesthetist	86.4% (38)	97.9% (47)	11.5%
Name of the operative procedure	84.1% (37)	97.9% (47)	13.8%
Type of incision	77.3% (34)	95.8% (46)	18.5%
Operative diagnosis	18.2% (8)	97.9% (47)	79.7%
Operative finding	90.9% (40)	95.8% (46)	4.9%
Complications encountered	15.9% (7)	93.8% (45)	77.9%
Any extra procedure performed with reason	6.8% (3)	93.8% (45)	87%
Details of tissue removed, added, or altered	25% (11)	93.8% (45)	68.8%
Details of closure technique	88.6% (39)	95.8% (46)	7.2%
Anticipated blood loss	2.3% (1)	95.8% (46)	93.5%
Antibiotic prophylaxis	2.3% (1)	97.9% (47)	95.6%
DVT prophylaxis	0% (0)	60.4% (29)	60.4%
Detailed post-operative care instructions	84.1% (37)	91.7% (44)	7.6%
Signature	95.3% (41)	100% (48)	4.7%
Legibility	93.2% (41) were satisfactory, 6.8% poor (3)	100% (48) were satisfactory	6.8%

Forty-eight operative notes were evaluated prospectively in the re-audit conducted in May-June 2022 (Table 1). Significant improvements were noted in several parameters in this re-audit. The date of procedure, type (emergency/elective), name of surgeon/assistant, and signature were documented in all the notes while operative diagnosis and antibiotic prophylaxis were documented in 97.9% (47) of the notes. DVT prophylaxis was documented in 60.4% (29) of the notes. All 48 notes were deemed to be satisfactorily legible. Overall, 94.3% of all parameters were found to be documented in the re-audit, which represents a 35.7% increase from the initial audit.

## Discussion

In our observations, we found that in the initial audit, 58.6% of the evaluated parameters were documented in operative notes in the Department of Surgery in Arif Memorial Hospital, Lahore. This increased to 95.3% in the re-audit that was conducted one month after the initial audit.

**Figure 1:**
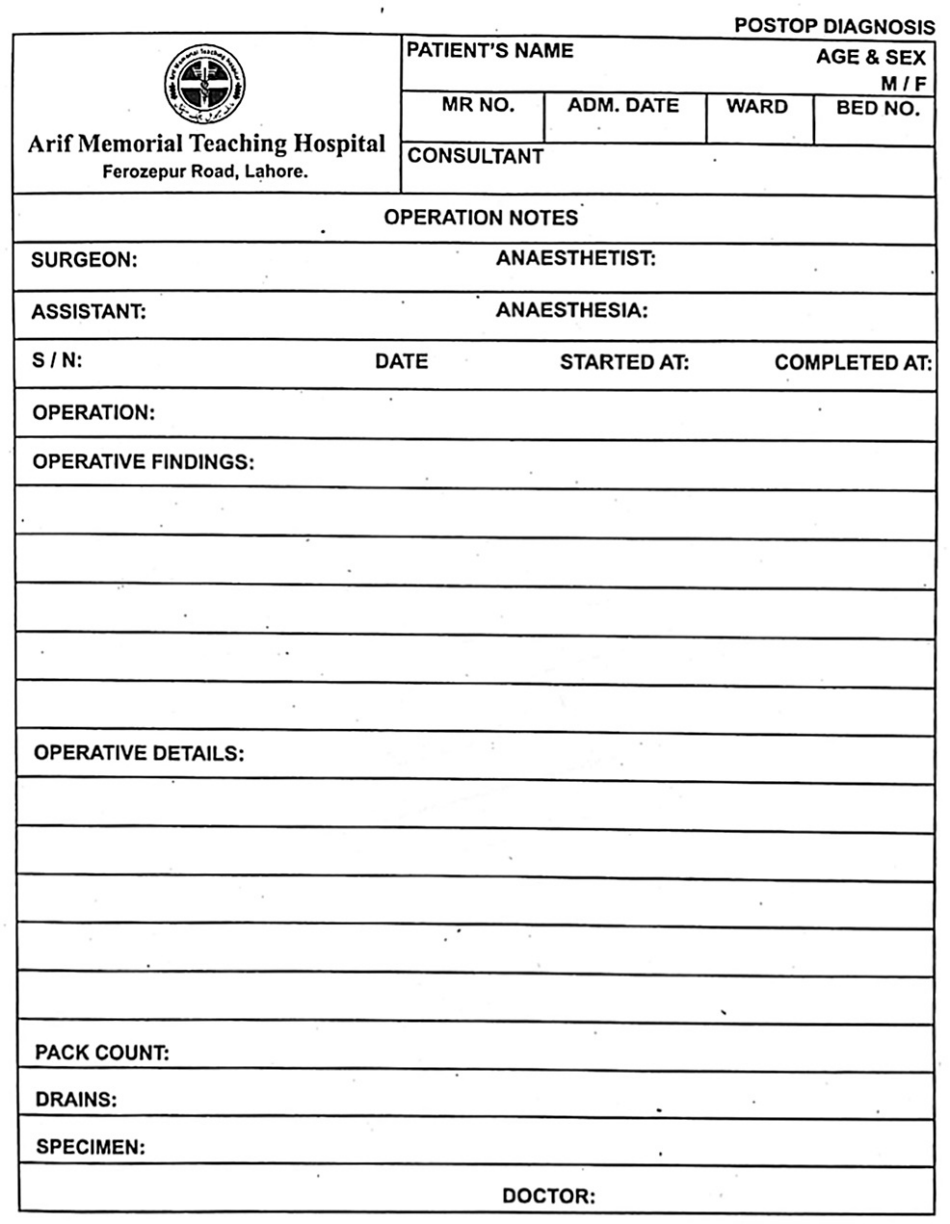
Operative notes pro forma, which were used before implementation of recommendations given after the initial audit.

**Figure 2:**
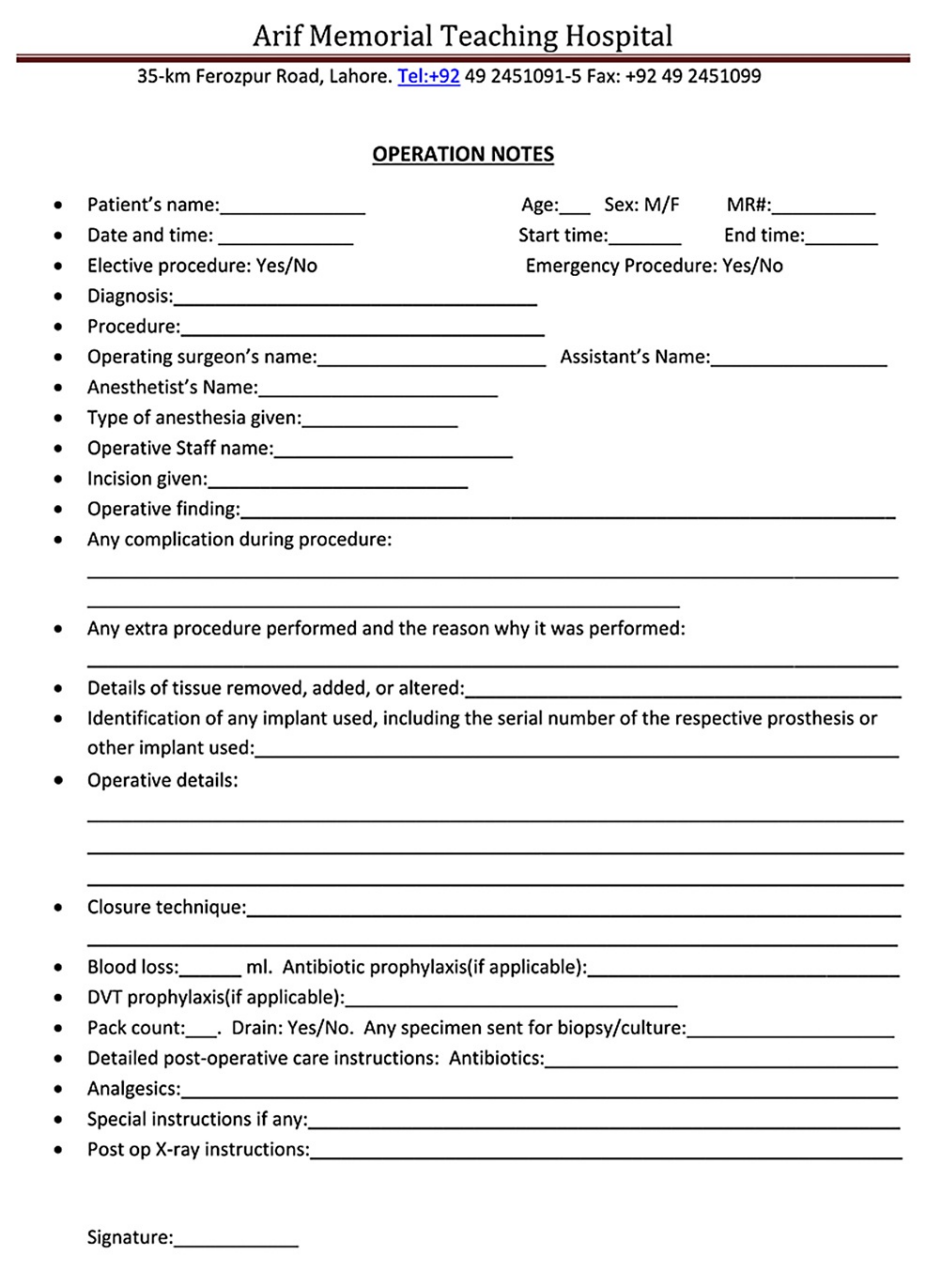
Redesigned operative notes pro forma, which were used for documentation after the initial audit. DVT: deep vein thrombosis.

The initial audit of operative notes highlighted several shortcomings in the process of documentation of these notes. These included inadequacies in the operative notes pro forma (Figure 1) and a lack of orientation regarding standards according to which these notes need to be documented. After the initial audit, the findings were presented to the Department of Surgery and several recommendations were made. The pro forma for operative notes was redesigned to include a section for several parameters, which were absent in the initial notes (Figure 2). These included DVT and antibiotic prophylaxis, type of procedure (emergency/elective), and a separate section for complications. A recommendation was also made to consider electronic medical records for the purpose of operative notes documentation, and this has been shown to increase the efficiency of documentation [10]. A poster detailing good practice guidelines for operative notes documentation was displayed in the surgeons' room, and surgeons were encouraged to document surgical notes soon after the procedure to improve accuracy. These recommendations demonstrated a positive change as a 35.7% improvement was noted in the re-audit. Finally, it was recommended that this audit should be conducted every six months to ensure sustainability in practice.

Khan et al. conducted a similar study in 2010 at Civil Hospital Karachi [9]. They found that the names of the operating surgeon, the name of the procedure, and post-operative instructions were mentioned in 99%, 99%, and 89% of the notes, respectively. This high percentage is consistent with our findings, which is likely due to the common template of pro forma used in public sector hospitals in Pakistan. Details of tissue removed and complications encountered were mentioned in a few operative notes, which is similar to our study and likely due to the shortcoming in the pro forma as well. However, in contrast to their study, our study showed a high percentage of documentation of the time of the procedure (96%) and details of the closure technique (87%).

Our study showed that none of the operative notes mentioned DVT prophylaxis in the initial audit, a finding that was consistent with the study conducted by Khan et al. [9]. Upon revision of the pro forma, the audit showed that 60.4% of the notes documented whether the patient needed DVT prophylaxis. In their audit, SARCO et al. also found that DVT prophylaxis was documented in 48.8% of the notes, which is similar to our study. The possible explanation for this low rate of documentation is probably the nature of the decision of prophylaxis itself. Generally, surgeons are more comfortable taking this decision after the immediate post-operative period, depending upon the bleeding status and general condition of the patient. This could be the reason that surgeons are hesitant to document instructions regarding DVT prophylaxis in the post-operative notes.

Clinical governance has taken center stage in health practices in the 21st century. Quality improvement projects are deemed essential to ensure the provision of good quality healthcare. In our closed loop audit, we found that testing practices against standards and subsequent re-auditing lead to significant improvement in practices. Similar results were noted by other authors who have found that documentation of operative notes significantly improved in the re-audit as compared to the initial audit [4,5,7,8].

## Conclusions

Our study showed significant improvement in the quality of operative notes after the implementation of recommendations following the initial audit of operative notes in Arif Memorial Hospital, Lahore. Such audits should be conducted routinely in all surgical departments to maintain and improve standards of documentation of operative notes.
